# The Hunterian Oration, Delivered in the Theatre of the Royal College of Surgeons in London, on the 14th of February, 1837

**Published:** 1837-07

**Authors:** 


### PART SECOND.

### Bibliographical Notices.

---

Art. I.—*The Hunterian Oration, delivered in the Theatre of the Royal College of Surgeons in London, on the* 14*th of February*, 1837. By Sir Benjamin C. Brodie, Bart. f.r.s. &c.—*London*, 1837. 8vo. pp. 38.

Compositions of this kind do not demand elaborate critical notice, but the one before us is by no means of a commonplace character. The Oration, as many of our readers doubtless know, was founded by Sir Everard Home and Dr. Baillie, in commemoration of John Hunter and of others who, like him, but in a less degree, contributed to advance the sciences connected with medicine. Unlike the annual formality at the College of Physicians, the coldness and dulness of which none can forget who have ever formed part of the audience of some thirty or forty melancholy individuals, assembled to gaze on the robed dignitaries of that establishment, and to hear names, before unknown to fame, enumerated in the list of the great deceased, the Oration at the College of Surgeons is in a language familiar to living men. The Oration at the College of Physicians is sometimes, we fear, to be ranked with those compositions, "pseudo-latinos, sed ferreo adeo et intorto sermone contextos, et tot barbaris sesquipedalibusque vocabulis horridos;" of which our sometime honoured teacher used to say, that neither ancients nor moderns, nor Scaliger himself, although he rose from the dead, could easily interpret them.

Of John Hunter's Life our pages contain so full an account, that we shall not refer to that part of the Oration which relates to it; but the rise of two such remarkable men as William Cullen and William Hunter from the obscurity of a small country town to the most distinguished stations in the profession, in the capitals of Scotland and England, is strikingly introduced; and the notice of William Hunter, especially, is as interesting as we believe it to be faithful.

"A century has just elapsed since a young man, established as a medical practitioner in the then small town of Hamilton, in Scotland, received into his house another young man, not many years junior to himself, as a pupil, that he might instruct him, as far as his limited means gave him the opportunity of doing so, in the elements of medicine and surgery. After the lapse of three years, an intimate friendship having become established between the master and the pupil, it was agreed that they should enter into a partnership, to practise in Hamilton as surgeons and apothecaries. For this purpose, however, it was thought necessary that the younger of these individuals should visit the medical schools, which had then been only lately established, in Edinburgh and London, so as to complete his education. Accordingly we find him, at the end of another year, studying anatomy in London under Dr. Douglas, a celebrated anatomist of that day, and filling at the same time the office of preceptor to Dr. Douglas's children. Here new schemes of life were offered to his ambition; and the result was, that he never returned to Hamilton. Not long afterwards his friend followed his example, seeking a wider field for the exercise of his talents, first in Glasgow, afterwards in Edinburgh. Of these young men the one, and the elder, was William Cullen, and the other was William Hunter: and such was the humble origin of two of the most remarkable men who ever engaged in the pursuit of the medical profession.

"Of Cullen, you well know that his talents raised him ultimately to the high situation of professor of medicine in the university of Edinburgh; and that, whatever may be the estimate which we now form of his pathological doctrines, they had a most extended influence, not only at the time of their being promulgated, but long afterwards; and that his system of nosology has, even within these few years, been a principal textbook of the medical schools.

"William Hunter, transplanted to London, entered enthusiastically into his new pursuits; and we find him, some time afterwards, writing to his friend Cullen in the following terms: 'Well! how does the animal economy appear to you, now that you have examined it, as one may say, with precision? I have good reason to put the question to you, because, in my little attempts that way, since I began to think for myself, Nature, where I am best disposed to mark her, beams so strong upon me that I am lost in wonder, and count it sacrilege to measure her meanest features by my largest conceptions.' Not many years elapsed before he became well known as a lecturer on anatomy. This was the foundation of his fortune; but he was ultimately recognized as one of the greatest pathologists at that time in Europe.

"I am not aware that there is any one present of such an age as to remember what William Hunter was as an anatomical teacher. But tradition supplies the place of memory; and I have, in the early part of my life, so frequently heard him spoken of in that capacity by older persons, that it seems to me almost as if I had been myself his pupil. He is reported to have been at once simple and profound; minute in his anatomical demonstrations, yet the very reverse of dry and tedious. Subjects, which were uninteresting in themselves, were rendered interesting by the liveliness of his descriptions; and the more important points were illustrated by the relation of cases and the introduction of appropriate anecdotes, which, while they relieved the painful effort of attention, served to impress his lessons on the mind in such a manner that they could never be effaced. His paper on the Structure of the Cartilages of Joints, published in the Philosophical Transactions for the year 1743, (at which time he was only twenty-five years of age, and in which he anticipated all that Bichat wrote sixty years afterwards respecting the structure and arrangements of the synovial membranes,) and his illustrations of the Gravid Uterus, sufficiently shew how correct he was in matters of detail, and at the same time how comprehensive were his general views. But we have evidence that his Lectures possessed merits of a higher order than these. His paper on the Uncertainty of the Signs of Murder in the Case of Bastard Children, published in one of the volumes of the Medical Observations and Inquiries, seems to have been little else than a transcript of a part of one of his lectures; and it is impossible to peruse it without being struck, not only with the intellectual penetration, the great good sense, and the power of argument, which is there displayed; but also with the indications which it affords of a humane, charitable, and even tender disposition. If we may venture, from this specimen, to form our judgment as to his other lectures, their tendency must have been to improve his pupils with respect to their moral qualities, fully as much as with respect to their professional attainments.

"It is natural for a man to delight in that occupation in which he is conscious that he excels; and accordingly we find that the delivery of his lectures on anatomy was William Hunter's favorite pursuit. At first he found it convenient to teach anatomy, as affording him the means of subsistence; but he continued to do so when the more lucrative pursuits of private practice had given him wealth beyond the most sanguine expectations of his early life. From this time, as I have been informed on good authority, he was accustomed to say, 'I wish to make no profit of my lectures; I am quite satisfied if they pay their own expenses;' which, of course, included those of the anatomical department of his museum. The performance of his duties as a lecturer was terminated only by his death; and I have been informed that, when his last moments had arrived, his mind still reverted to that which he regarded as the most worthy occupation of his life, and that he said 'I wish now that I had but strength to bear being carried into my theatre, that I might tell my pupils how much comfort and happiness I feel.'" (P. 8.)

Sir Benjamin adds, that he could almost wish that the Commentaries of this great physician had never been published, displaying, as they do, such marks of weakness, and of unnecessary anxiety on the subject of his well-founded reputation as a scientific discoverer.

In our notice of Mr. Ottley's Life of John Hunter, we alluded to the extraordinary fact that about 70,000*l.* were expended by that great physiologist on his museum. William was equally munificent in the same way. His museum and collection, now at Glasgow, is said to have cost 100,000*l.*; and all this was saved, in the instance of both brothers, from their professional emoluments. This noble collection, which it is the just pride of the ancient college of Glasgow to possess, might have been part of a vast national museum in London, but for the indifference of the government of the day to all scientific matters.

Among those mentioned with honour in the Oration, as Hewson, Cruikshank, &c., the features of the late excellent Dr. Baillie's character are well and justly portrayed: "those great and good qualities which obtained for him, in an unusual degree, the respect and esteem of his profession; his sagacity, his knowledge, his judgment; his sincerity, his consideration for the feelings of others, and the total want of selfishness, or what may be better expressed by a phrase drawn from another language, the *abandonnement de soi,* which he exhibited on all occasions." (P. 29.) Sir Everard Home is spoken of with the gratitude of one indebted to him for acts of kindness in the early part of the author's career; and Sir William Blizard is kindly remembered.

It is mentioned that the library of the College now contains not fewer than 20,000 volumes, and is open not only in the early part of the day, as formerly, but on three evenings in a week; that a *catalogue raisonné* is in preparation; and that the lectures of Mr. Owen, the junior conservator, on anatomy and physiology, will contribute to form "a school of what may be called 'the science of life,' such as has never existed in the metropolis before:" all which is very gratifying. It is clear that the surgeons will slumber no more; but when will the physicians awake?

